# Magnetite Oxide Nanomaterial Used for Lead Ions Removal from Industrial Wastewater

**DOI:** 10.3390/ma14112831

**Published:** 2021-05-25

**Authors:** Oana Stoian, Cristina Ileana Covaliu, Gigel Paraschiv, Gina-Alina Catrina (Traistaru), Mihai Niță-Lazăr, Ecaterina Matei, Sorin Ștefan Biriş, Paula Tudor

**Affiliations:** 1 University Politehnica of Bucharest, 313 Splaiul Independentei, 060042 Bucharest, Romania; stoian_s_oana@yahoo.com (O.S.); paraschiv2005@yahoo.com (G.P.); ecaterinamatei@yahoo.com (E.M.); biris.sorinstefan@gmail.com (S.Ș.B.); paulavoicu85@yahoo.com (P.T.); 2 National Research and Development Institute for Industrial Ecology-ECOIND, 71-73 Drumul Podu Dambovitei Str., 060652 Bucharest, Romania; traistaru_ginaalina@yahoo.com (G.-A.C.); mihai.nita@incdecoind.ro (M.N.-L.)

**Keywords:** heavy metal, lead ions, adsorption, nanotechnology, wastewater treatment

## Abstract

The aim of this article is to present a nonconventional method for the efficient removal of lead ions from industrial wastewater. For this purpose, magnetite nanomaterial was used, which was very easily separated from the wastewater at the end of the treatment due to its magnetic properties. Currently, nanotechnology is an efficient and inexpensive manner that is being researched for wastewater treatment. Additionally, iron oxide nanoparticles are widely used to remove heavy metal ions from water due to their special properties. The experimental results detailed in this article show the influence of pH and contact time on the process of adsorption of lead ions from wastewater. The magnetite nanomaterial had its maximum efficiency of speed when the wastewater had pH 6. At a lower pH, the highest treatment efficiency was over 85%, and the required contact time has doubled. When the pH increases above 6, the precipitation process occurs. Langmuir and Freundlich models were used to describe the adsorption process.

## 1. Introduction

Various methods for removing lead have been used, including adsorption, coprecipitation [1], reverse osmosis [2], ion exchange [3], membrane filtration [4], etc. Among these, the most preferred method is adsorption, since it is simple, economical, and environmentally friendly.

Nanotechnology is used for environmental remediation because it can provide a potentially cheap and efficient way for wastewater treatment [5]. Magnetic nanoadsorbents such as spinel ferrites, maghemite, and hematite are strong adsorbents for the removal of pollutants from wastewater. The application of an external magnetic field will easily isolate them from the reaction media due to their magnetic properties. Additionally, the application of magnetic separation on the nanoadsorbents provides the invaluable benefit of the rapid recovery of toxic metals from wastewater [6]. Due to their easy separation from wastewater and low toxicity, iron oxide nanoparticles are commonly used for metal removal [7,8]; in addition, if the nanoparticles are composed of magnetite, they can be easily separated from the associated pollutants [9]. The literature shows that magnetite nanoparticles are of a wide variety and can be further modified to improve their properties. The literature shows numerous studies on the use of magnetic nanoadsorbents to remove specific heavy metals in their ionic states, such as chromium, nickel, arsenic, cobalt, lead, copper, and others [10,11,12,13,14,15]. Magnetite nanoparticles are a promising solution because they are hydrophilic, are super paramagnetic, and have a high surface area [16]. The efficient implementation of magnetic nanoadsorbents depends on their efficiency in the selective adsorption of the pollutants involved and their surface chemistry [6,15].

**Figure 1:**
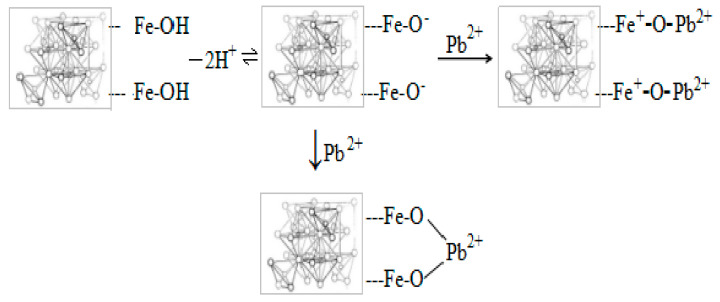
The adsorption mechanism of lead on magnetite nanoparticles [17].

The potential process of lead ion adsorption on magnetite nanoparticles is shown in Figure 1. On the surface of the magnetite nanoparticles, certain surface groups are formed, namely -FeOH^2+^ and -Fe^+^, due to protonation and deprotonation. It is observed that -Fe-O- may easily bond with Pb^2+^.

The advantages of the adsorption method using magnetic nanomaterials, including magnetite, are their high efficiency of removal of heavy metal ions such as Pb(II), avoidance of secondary waste generation, production of no secondary pollutants, ability to treat large amounts of wastewater in a short time, good selectivity, and potential to be easily recycled and utilized on an industrial scale [18]. Due to their smaller size, magnetite nanomaterials have an increased surface area, thus improving adsorption capacities for lead ion removal.

Lead is one of the heavy metals that is associated with toxic poisoning even if it exists in low concentrations in wastewater. According to NTPA 001/2002, the maximum value of lead ions in wastewater must not exceed 0.20 mg/L. In industrial wastewater, lead ions are found in concentrations of about 200–500 mg/L; the concentrations are very high in relation to the values imposed by the legislation in force [19]. The main sources of lead ion pollution are effluents from battery processing, steel industries, fuels, paint pigment, photographic materials, automobiles, aeronautics, explosives manufacturing, or coating industries [20,21]. Accumulation of lead ions in the human body can lead to cancer, memory problems, brain damage, high blood pressure, kidney disease, premature birth, hearing loss, or low IQ in children [22,23,24].

In order to regenerate the magnetite nanoparticles, the researchers noticed that an acidic environment is needed [25,26]. Thus, a high regeneration efficiency using 0.1 M H^+^ was obtained, and the adsorption capacity of the reused magnetic nanoparticles remained almost constant over the next four cycles.

In order to remove lead ions from wastewater, the researchers used magnetite nanoparticles or composite nanomaterials that contain magnetite nanoparticles. Examples of the materials used in the lead ion adsorption process, which includes working conditions and treatment efficiencies, are recorded in Table 1.

In the present study, the adsorption process was applied in order to remove lead ions from wastewater. This article provides novel research through the following considerations: the utilization of magnetite nanoparticles with different characteristics [27] from other magnetite nanoparticles used in the adsorption process of lead ions from wastewater [6,28,29], testing wastewater samples with different concentrations of lead ions (0.70, 1.00, 1.20, 1.45, 1.64 mg/L) from the scientific literature, and conducting experiments with two temperature values.

## 2. Materials and Methods

In this study, the nanomaterial used to remove lead ions from synthetic wastewater was magnetite (Fe_3_O_4_). This was obtained through the coprecipitation process. The magnetite particles applied in the process of adsorption of lead ions from wastewater were synthesized using a concentration of aqueous sodium hydroxide solution of 0.8 mol/L. The molar ratio of Fe^2+^: Fe^3+^: PEG: PVP unit was set at 1: 2: 3: 4. Centrifugation was used to separate the precipitate, and then it was washed several times with water. Precursor calcination was performed at 410 °C for a period of 2 hours to obtain Fe_3_O_4_ powder [27]. The reagents that were used to determine the concentration of lead ions (hydroxylammonium chloride, ammonia solution, and potassium cyanide) were purchased from Sigma-Aldrich, Burlington, MA, USA. The analysis of lead ion concentrations in synthetic wastewater was performed with the PhotoLab S12 photometer purchased from Wissenschaftlich-Technische Werkstätten GmbH (WTW), Weilheim, Germany.

All experiments were performed at room temperature and at 30 °C with rotational speeds of 300 and 400 RPM having different concentrations (0.70; 1.00; 1.20; 1.45; 1.64 mg/L), and the dose of adsorbent nanomaterial was 2.00 g/L. The pH values were set at 4 and 6 for each concentration using 0.1 M HCl. The experimental results were obtained in accordance with ISO 8466-1 and DIN 38402 A51 (10-mm cell) with a measuring range between 0.010 and 5.00 mg/L Pb (II).

The equilibrium adsorption amount (q_e_, mg/g) of Pb (II) ions was calculated using the following formula:

(1) $$q_{e}=\frac{(C_{i}-C_{e})\times V}{W}.\;$$

where C_i_ is the initial concentration of Pb (II) ions (mg/L)

C_e_ is the equilibrium concentration of Pb (II) ions (mg/L)

V is the volume of the solution (L)

W is the amount of the nanomaterial (g).

Furthermore, the treatment efficiency (ƞ, %) was also determined by the following equation:

(2) $$ƞ=\frac{C_{i}-C_{f}}{C_{i}}\times 100$$

where Ci is the initial concentration of Pb (II) ions (mg/L)

Cf is the final concentration of Pb (II) ions (mg/L).

In this article, two models of adsorption isotherms have been described in order to remove lead ions from wastewater, namely Langmuir and Freundlich.

The formula used to describe the Langmuir model is presented below:

(3) $${\text{q}}_{\text{e}}=\frac{{\text{Q}}^{0}\times {\text{K}}_{\text{L}}\times {\text{C}}_{\text{e}}}{1+{\text{ K}}_{\text{L}}\times {\text{C}}_{\text{e}}}$$

where q_e_ is the quantity of Pb (II) adsorbed by magnetite at equilibrium (mg/g)

Q_0_ is the maximum monolayer coverage capacity (mg/g)

K_L_ is the Langmuir isotherm constant (L/mg)

C_e_ is the equilibrium concentration of adsorbate (mg/L).

The R_L_ parameter was calculated using the following formula:

(4) $$R_{L}=\frac{1}{1+\left(1+K_{L}\times C_{0}\right)}$$

where R_L_ is the value that indicates if the process is unfavorable (R_L_ > 1), linear (R_L_ = 1), favorable (0 < R_L_ < 1), or irreversible (R_L_ = 0) [22]

K_L_ is the Langmuir constant

C_0_ is the initial concentration [mg/L].

The Freundlich model is followed using the equation below:

(5) $$q_{e}=K_{F}\times C_{e}{}^{1/n}$$

where q_e_ is the quantity of metal adsorbed by magnetite at equilibrium (mg/g)

K_F_ is the Freundlich isotherm constant (mg/g)

C_e_ is the equilibrium concentration of adsorbate (mg/L)

n is the adsorption intensity.

## 3. Results

### 3.1. The pH Effect

**Figure 2:**
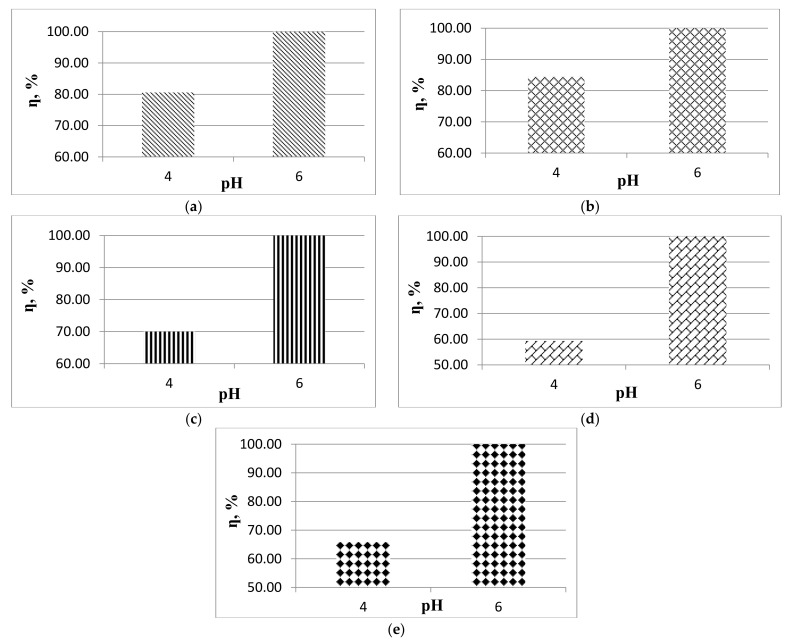
The pH effect on the removal process of Pb (II) ions (conditions: metal ion concentration 0.70 (**a**); 1.00 (**b**); 1.20 (**c**); 1.45 (**d**); 1.64 (**e**) mg/L).

The removal efficiency of lead ions from wastewater was performed at two pH values, 4 and 6. The results can be seen in Figure 2.

**Figure 3:**
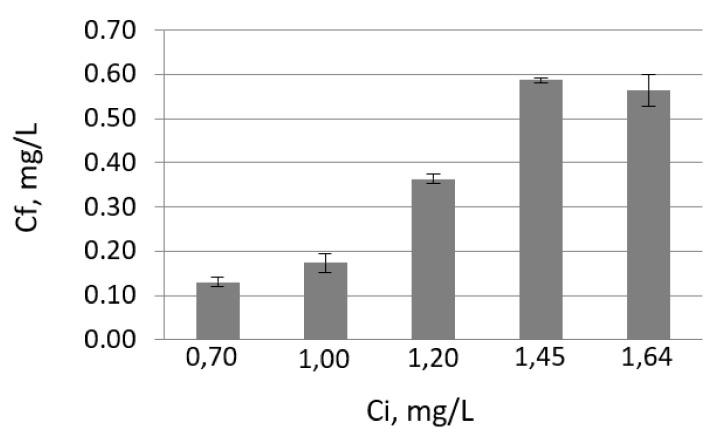
Error bars for experiments performed at pH 4.

The experiments were done in triplicate. Thus, the error bars are presented in Figure 3 in the case of experiments performed on wastewater with pH 4.

It can be seen that in the case of pH value 4, the treatment efficiencies reach up to 84.40% in the case of the removal of a concentration of 1.00 mg/L of lead ions from the wastewater, while, at pH value 6, 100% treatment efficiencies were obtained. It is also observed that with the increase of the initial concentration of lead ions, the required contact time increases more in the case of pH 4 (from 180 to 420 min) than in the case of pH 6 (from 90 to 270 min). Moreover, at pH 6, the process is faster (up to 270 min) than at pH 4 (up to 420 min). The experiments performed at pH values greater than 6 do not show certainty that lead ions will be removed from the synthetic wastewater solution by the adsorption process.

In the case of the interaction of lead ions with the magnetite nanomaterial, the literature shows that in case of pH higher than 6.5 Pb(OH)_2_, it is the dominant species, and in case of pH lower than 6.5, we have Pb^2+^ and Pb(OH)^+^ [45]. The reaction below demonstrates that the surface of the adsorbent nanomaterial can be subjected to protonation or deprotonation [46,47]:

(6) $$H_{2}O+M-O^{-}<\frac{H^{+}}{{\mathrm{OH}}^{-}}>M-\mathrm{OH}<\frac{H^{+}}{{\mathrm{OH}}^{-}}>M-{\mathrm{OH}}_{2}^{+}$$

Once the wastewater pH is basic, there will be a fairly high electrostatic attraction between the surface of the magnetite nanomaterial that is negatively charged and lead ions. When the pH of the wastewater decreases, the number of sites that are positively charged will increase, and the number of sites that are negatively charged will decrease. Thus, the adsorption of lead ions is not favored due to electrostatic repulsion. In an acidic environment, hydrogen ions that are present in excess will compete with lead ions for adsorption sites, and therefore the treatment efficiency is lower.

In water, magnetite nanoparticles present surface hydroxyl groups (Fe–OH). Depending on the pH of the wastewater, the protonation or deprotonation of the hydroxyl groups is observed. At pH < pH_pzc_ the surface of the nanoparticles is positively charged (FeOH_2_^+^), and at pH > pH_pzc_ the surface of the nanoparticles is negatively charged (FeO^−^) [28,48,49]. As the pH of the wastewater increases from 4.0 to 6.0, the adsorption of lead ions increases due to the electrostatic attractions that occur between Fe–O and Pb^2+^. The low adsorption of lead ions at pH 4 is due to the competition for adsorption sites on the surface of available magnetite nanoparticles between H_3_O^+^ and Pb^2+^. The inner surface of magnetite nanoparticles forms a complex that has a covalent bond between the lead ion and the surface oxide’s oxygen.

### 3.2. Contact Time Effect

**Figure 4:**
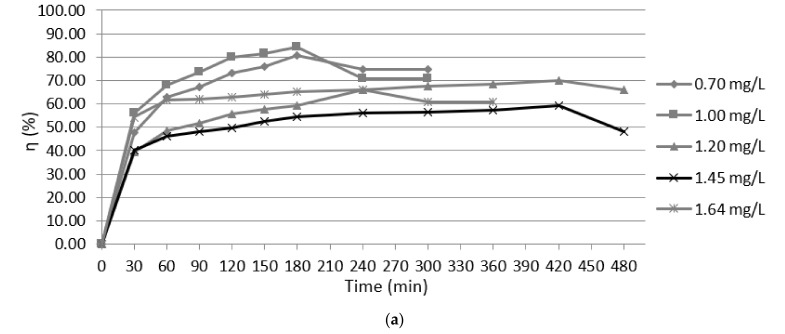

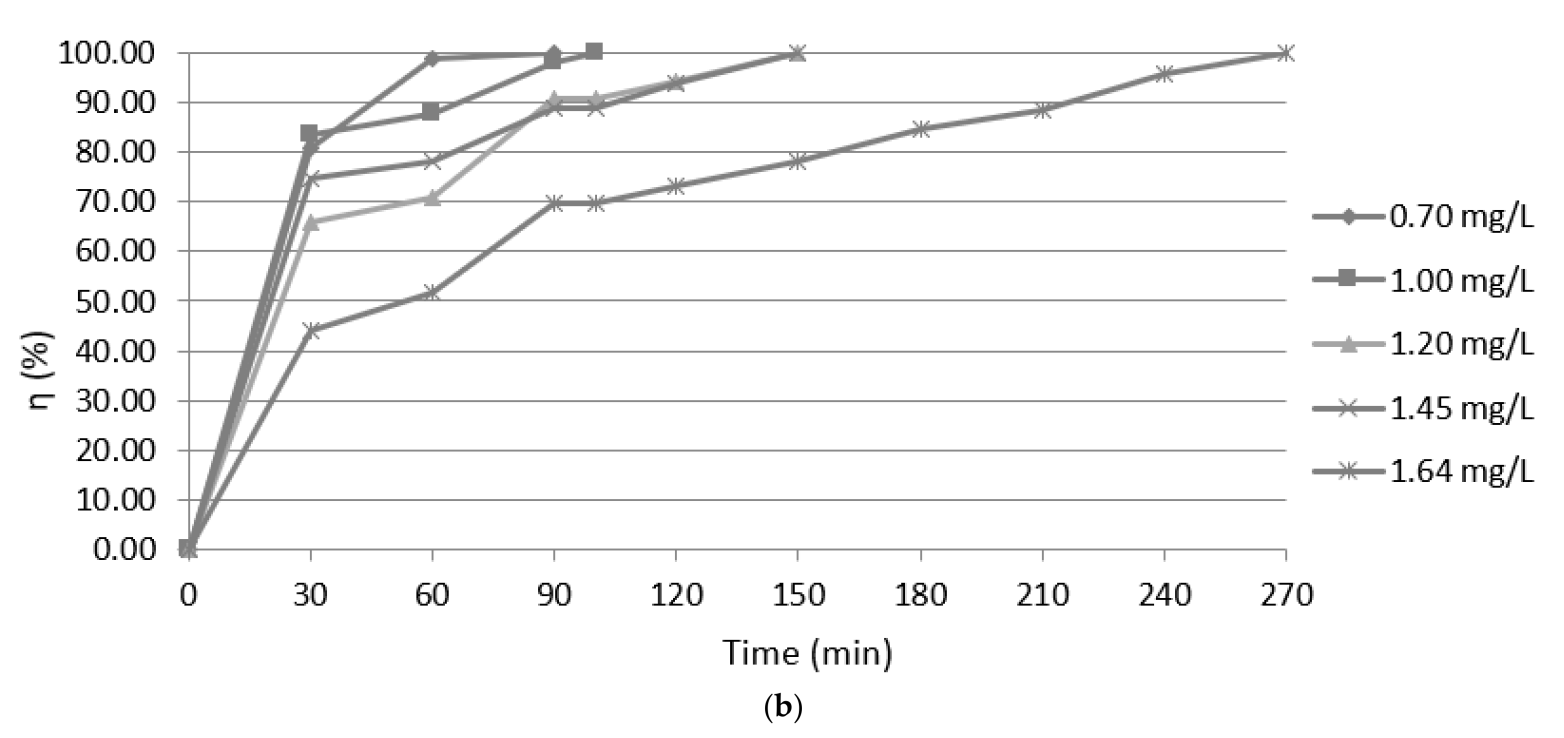
Efficiencies of wastewater treatment versus time at pH 4 (**a**) and pH 6 (**b**).

The effect of contact time on wastewater at pH 4 and 6 was studied and can be seen in Figure 4.

From Figure 2 and Figure 4, we can see that the concentration of lead ions decreases for a while, then increases. Thus, the treatment efficiency increases up to a certain percentage, then decreases. In the case of pH 6, the equilibrium concentration (90 min for the initial concentration 0.70 mg/L, 150 min for the initial concentration 1.20 mg/L) was reached in a shorter time than in the case of pH 4 (180 min for the concentration 0.70 mg/L, 420 min for the initial concentration 1.20 mg/L). In the first 60 min (at pH 4) and 30 min (at pH 6), the removal of lead ions from wastewater is faster due to the rapid occupation of the sites on the surface of the magnetite.

### 3.3. The Impact of Temperature on the Adsorption Process

**Figure 5:**
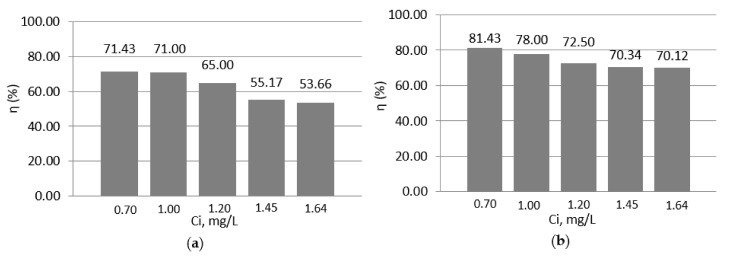
Efficiencies of wastewater treatment at 30 °C versus initial concentrations at pH 4 (**a**) and pH 6 (**b**).

To observe the effect of temperature on the adsorption process of lead ions on the magnetite nanomaterial, the temperature was increased to 30 °C, and the results are shown in Figure 5.

In this case, increasing the temperature to 30 °C leads to lower treatment efficiencies than in the case of the experiments performed at room temperature. In the case of pH 4 and 6, the difference between the two temperatures is between 4.14 and 13.4% and 18.57 and 29.88%, respectively, depending on the initial concentrations of the pollutant.

### 3.4. The Impact of Rotation Speed on the Adsorption Process

**Figure 6:**
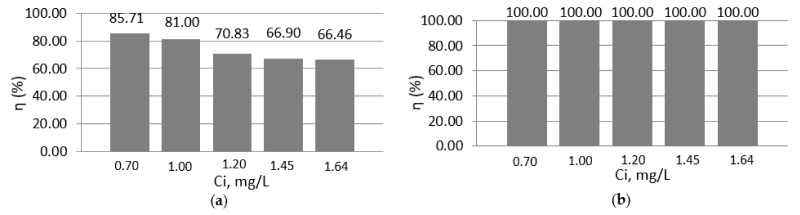
Efficiencies of wastewater treatment in the case of 400 RPM versus initial concentrations at pH 4 (**a**) and pH 6 (**b**).

The rotation speed was increased to 400 RPM to see if there was an improvement in the treatment efficiencies; the results can be found in Figure 6.

When the rotational speed was increased from 300 RPM to 400 RPM the results were clearer and increased slightly, the difference between the treatment efficiencies reaching up to a percentage of 7.59% depending on each initial concentration of lead ions. Apart from this aspect, the treatment time was shortened by up to 30 min. In the case of pH 6, the treatment efficiencies also reached 100%.

### 3.5. Adsorption Isotherms

**Figure 7:**
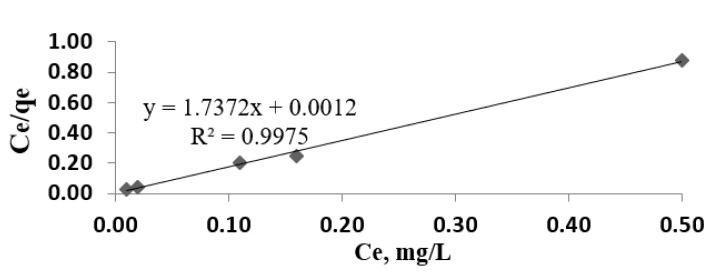
Experimental isotherm for different concentrations of lead ions (0.70; 1.00; 1.20; 1.45; 1.64 mg/L).

**Figure 8:**
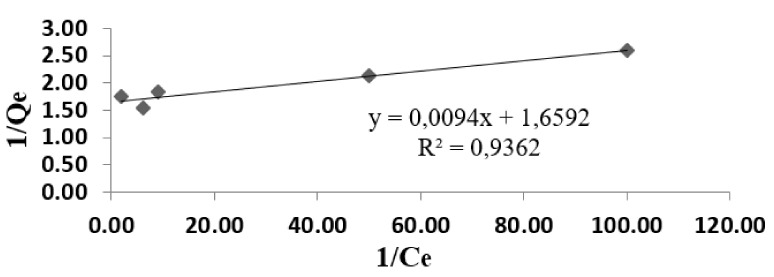
Langmuir model for the adsorption of lead ions from wastewater.

**Figure 9:**
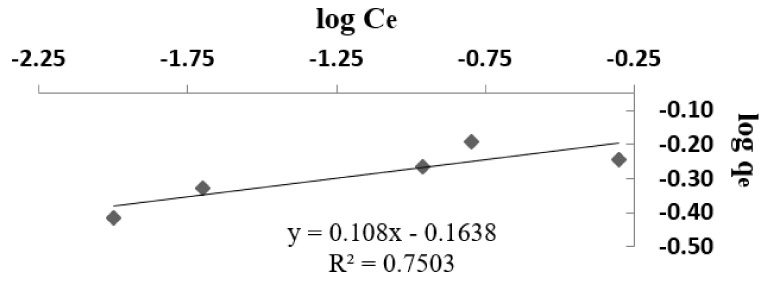
Freundlich model for the adsorption of lead ions from wastewater.

The experimental data were used for Langmuir and Freundlich models (Figure 7, Figure 8 and Figure 9). Modeling of adsorption isotherms provides information about the adsorption process, the surface properties of the adsorbent nanomaterial, and its affinities. The constants of the Langmuir and Freundlich adsorption isotherms obtained from the processing of experimental data are presented in Table 2.

Due to the fact that the R_L_ value is between 0 and 1, it is understood that the adsorption is favorable and that there is an efficient interaction between the adsorbent nanomaterial Fe_3_O_4_ and lead ions in accordance with the Langmuir model.

The Freundlich isotherm explains the monolayer and multilayer adsorption. The values K_F_ and n can be determined using a linear equation resulting from plotting the curve log q_e_/log C_e_ (Figure 9). Thus, the value 1/n must be between 0 and 1 in order to apply the Freundlich model. In the present case, the value of 1/n is 0.10, thus indicating that the adsorption process is favorable.

## 4. Conclusions

In the case of wastewater with pH 6, the treatment efficiency reached 100% at all studied concentrations at room temperature. The highest treatment efficiency in the case of the removal lead ions from wastewater at pH 4 was 85.71% at the initial concentration of 0.70 mg/L at room temperature when the rotational speed was set to 400 RPM. The required contact time is longer in the case of pH 4 than in the case of pH 6. The minimum contact time at which the maximum treatment efficiency was reached was 60 minutes and 150 minutes in the case of wastewater with pH 6 (100%) and pH 4 (85.71%), respectively. There is also the phenomenon of desorption (reversible adsorption).

As the pH of the wastewater increases (up to pH 6), the adsorption of lead ions on the Fe_3_O_4_ surface is facilitated. On the other hand, if the pH rises above 6, the removal of lead ions can be achieved through the adsorption process, but precipitation can also occur due to the metallic hydrolysis of lead hydroxide.

Compared to the literature, in the case of the experiments presented in this article, the concentrations studied for lead ions were lower, but the importance of the pH of the wastewater was observed, and the treatment process was fast. The study of lower concentrations of lead ions in laboratory conditions demonstrates the maximum treatment efficiency, and the data can be reported on an industrial scale.

## Figures and Tables

**Table 1:** Materials used for lead removal from wastewater.

Adsorbent	Quantity of Adsorbent (g/L)	Solution Volume (mL)	pH	Contact Time (min)	Speed (rpm)	Temp. (°C)	C_i_ (mg/L)	Ƞ (%)	Ref.
Fe_3_O_4_	0.05	100	4	30	-	25	50	≈84.00	[6]
			6					≈85.00	
			9					≈95.00	
Fe_3_O_4_	1.00	50	5	-	-	25	25	91.00	[28]
							50	56.00	
							100	31.00	
Fe_3_O_4_	10.00	10	5.5	1440	200	25	220	100.00	[29]
Chitosan/magnetite	0.10	100	6	120	-	Room temp.	70	90.47	[30]
Magnetite (Fe_3_O_4_) nanospheres	1.00	50	5	-	-	25	10	>70.00	[31]
Fe_3_O_4_/cyclodextrin polymer	12.00	10	5.5	120	230	25	100	99.50	[32]
PVP–Fe_3_O_4_	-	-	6.5	90	200	-	1	100.00	[9]
15% Fe_3_O_4_/SiO_2_	5.00	25	4.8	360	-	Room temp.	50	99.84	[33]
Fe_3_O_4_ @ SiO_2_–NH_2_ core-shell	1.00	50	5.2	960	-	25	148	≈87.83	[34]
L-cysteine functionalized Fe_3_O_4_	1.00	50	6	60	200	25	50	45.00	[16]
	2.00							99.00	
	2.50							99.00	
Natural goethite	40.00	25	5	-	-	30	750	100.00	[35]
Peat moss	0.24	100	6	180	125	23±1	10	96.00	[36]
Waste beer yeast	20.00	-	5	120	150	-	25–100	96.34	[37]
Coal fly ash	1.50	50	-	240	-	-	100	90.37	[38]
Sawdust waste	2.00	50	6.5	240	200	30	103.6	88.60	[39]
Activated bamboo charcoal	1.00	100	5	360	150	29	60	83.01	[40]
Banana peels	40.00	-	5	20	100	25	50	85.30	[41]
Coconut shell	1.00	50	4.5	180	180	-	10	99.00	[42]
Natural orange peel	10.00	12	5	60	-	Room temp.	30	99.00	[43]
Ficus Religiosa leaves	10.00	100	4	45	200	50	100	80.00	[44]

**Table 2:** Adsorption isotherm constants.

Adsorbent	Langmuir Isotherm			Freundlich Isotherm			
Fe_3_O_4_	K_L_(L/mg)	R_L_	R^2^	1/n	n	K_F_ (mg/g)	R^2^
	0.60	0.68	0.93	0.10	9.25	1.45	0.75

## Data Availability

Data sharing is not applicable.
